# Conservative treatment of a young adult patient with a moderate skeletal Class III malocclusion by applying the temporary anchorage devices and the surgically assisted rapid palatal expansion

**DOI:** 10.1002/ccr3.1245

**Published:** 2017-10-30

**Authors:** Panpan Liu, Hui Chen, Xiaoxin Shi, Jing Guo

**Affiliations:** ^1^ Department of Pediatric Dentistry School & Hospital of Stomatology Shandong University Jinan China; ^2^ Department of Orthodontics School & Hospital of Stomatology Shandong University Jinan China; ^3^ Key Laboratory of Oral Biomedicine of Shandong Jinan China; ^4^ Department of Oral Radiology Academic Centre for Dentistry Amsterdam (ACTA) University of Amsterdam VU University Amsterdam Amsterdam The Netherlands; ^5^ Department of Oral Kinesiology Academic Centre for Dentistry Amsterdam (ACTA) MOVE Research Institute Amsterdam University of Amsterdam VU University Amsterdam Amsterdam The Netherlands

**Keywords:** Case report, Class III, micro‐implants, surgically assisted rapid palatal expansion

## Abstract

We present the orthodontic treatment of a 20‐year‐old Chinese man with the moderate skeletal Class III malocclusion. The usages of the temporary anchorage devices and the surgically assisted rapid palatal expansion (SARPE) have provided a variety of options for the treatment of Class III malocclusion.

## Graphical abstract

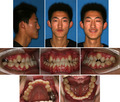

## Introduction

The treatment for the skeletal Class III malocclusion is among the most challenging for orthodontists. The adult patients with the skeletal discrepancy are often advised to conduct the treatment combining orthodontic and orthognathic surgery, which is usually unacceptable to patients owing to the potential risk and expenses. The anterior cross‐bite with the functional shift of the mandible is also called a pseudo‐Class III malocclusion 1, 2, which is caused by a forward functional displacement of the mandible owing to the premature occlusal interferences.

The maxilla of patients with Class III is usually insufficient in the transverse dimension 3. As the surgical technique of surgically assisted rapid palatal expansion (SARPE) involving the mid‐palatal suture split introduced in the first half of the 20th century, the technique has gone through significant evolution 4. It was previously recorded that the resistance to the maxillary expansion existed mainly in the mid‐palatal suture, zygomatic buttress, and pterygomaxillary sutures 5, 6, 7, 8. Whether to choose the surgical method to relieve the resistance of the maxillary expansion depends on the age of the patient.

The temporary skeletal anchorage devices were reported to be used to distalize the mandibular dentition to correct the Class III malocclusion 9, 10, 11. The micro‐implants could be placed in the interradicular space between the mandibular first molars and second molars or between the first molars and the second premolars or in the retromolar area 1, 10. The implantation in the retromolar area can allow more distal movement of the mandibular dentition. However, the handling procedures require more operational skills and the gingival irritation and inflammation of the surrounding soft tissue are easily induced, which usually result in the failure of the micro‐implants.

With the advanced treatment technology, more adult patients with Class III malocclusion prefer to choose the orthodontic conservative treatment. The purpose of this case report is to present a young adult patient with the moderate skeletal Class III malocclusion and the functional shift of mandible, who is treated with the fixed orthodontic appliances, SARPE, and mandibular micro‐implants.

## Case Presentation

**Figure 1:**
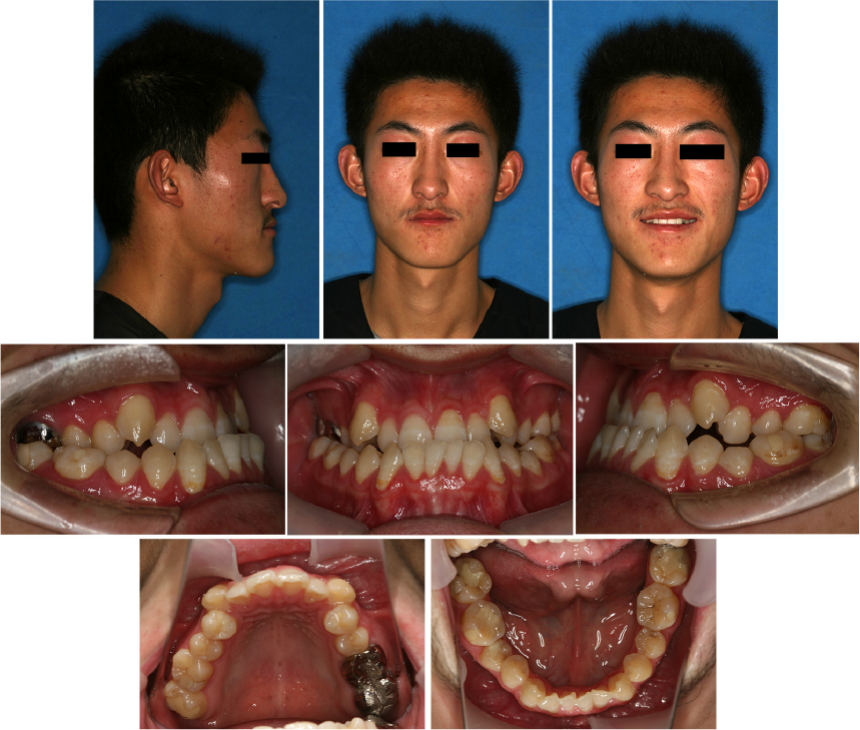
Pretreatment extra‐oral and intra‐oral photographs.

The patient was a 20‐year‐old Chinese man with the chief concern of the “inefficient chewing and unpleasant smile.” He had a concave, asymmetrical face, and a prognathic mandible with a Class III appearance (Fig. 1). His medical history was noncontributory. His dental history included the unqualified metal crowns on the maxillary right first and second molar. The carious lesions on the maxillary left canine, mandibular left second premolar, and the mandibular first and second molars had been treated.

The clinical examination showed that the patient had a concave profile with a relatively long lower face and a tendency of lower lip protrusion. The nasolabial angle was decreased, and the mentolabial sulcus was flat. From the frontal view, the face was slightly asymmetrical and the chin was deviated to the left. The mentalis was hyperactive when the lips were closed under compulsion. The display of the maxillary incisors was insufficient, and the smile arc was unesthetic in that maxillary incisal edges were nonconsonant with the lower lip curvature (Fig. 1).

**Figure 2:**
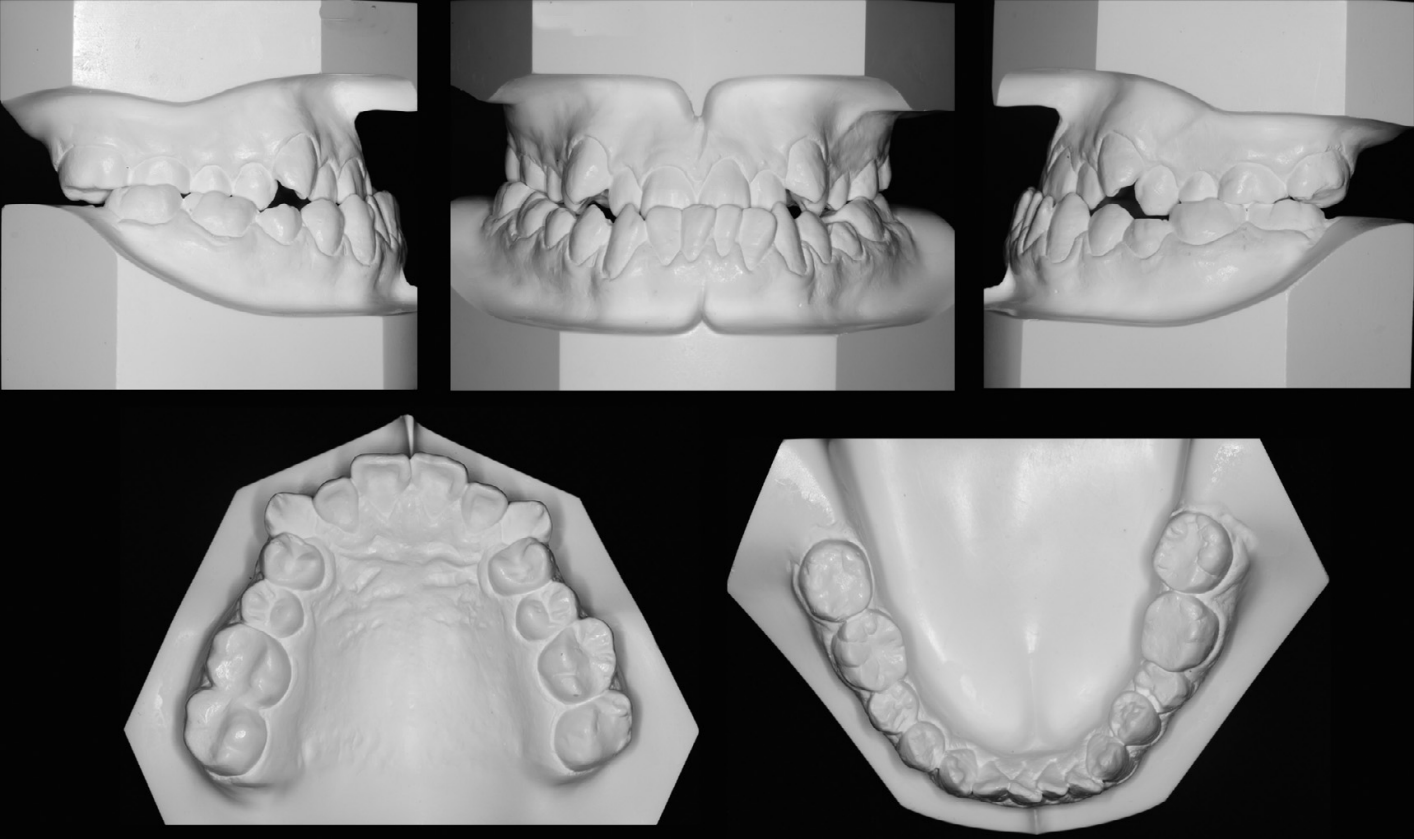
Pretreatment dental casts.

Intra‐orally, he had Class III canine and molar relationship on both sides. He had an overjet of −1.0 mm and a 30% reversed overbite. The arch length deficiencies in the maxillary arch were −8.0 mm and −7.0 mm in the mandibular arch. The maxillary canines were blocked out, and the mandibular canines exhibited the gingival recession. The full arch was in cross‐bite when the mandible was in centric occlusion. Comparing to his facial midline, the maxillary dental midline was deviated 1.0 mm to the right and the mandibular midline was 2.0 mm to the left, leading to a 3.0‐mm dental midline discrepancy. A functional shift of the mandible could be detected and when the mandible was guided into the centric relation, the incisors showed an edge‐to‐edge relationship (Figs 1 and 2).

**Figure 3:**
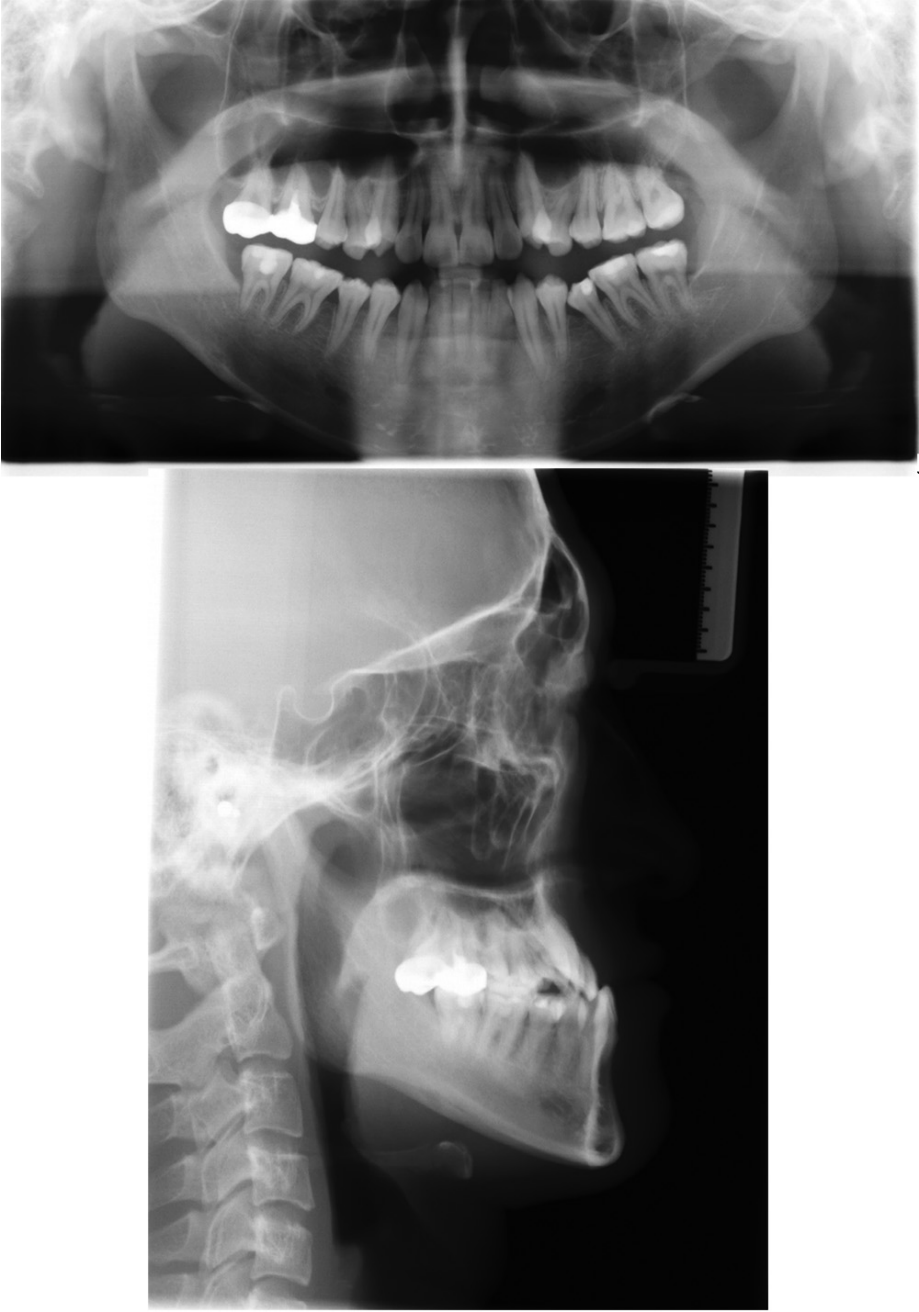
Pretreatment panoramic radiograph and lateral cephalogram.

The panoramic radiograph showed a full permanent dentition without the third molars, and the caries or pathologies on the maxillary left canine, mandibular left second premolar, and the four mandibular molars had been treated. The lateral cephalometric analysis indicated a skeletal Class III malocclusion (ANB, −3.5°; Wits appraisal, −15.0 mm) with an average growth pattern (SN‐MP, 29.5°; FH‐MP, 23.6°; S‐Go/N‐Me, 68.0%). The mandibular length was excessive comparing to the anterior cranial base (Go‐Me, 79.0 mm; S‐Na, 69.0 mm; Go‐Me/S‐Na, 0.87). The inclination of maxillary incisors was within normal range, and the mandibular incisors were lingual tipping (U1‐SN, 108.2°; IMPA, 72°). His sister also had a skeletal Class III pattern. Therefore, the etiology of his malocclusion may be a combination of genetic and environmental factors (Fig. 3; Table 1).

**Table 1:** Cephalometric measurements

Variable	Norm	Pretreatment	During treatment	Post‐treatment
Sagittal				
SNA (°)	82.8 ± 4.0	77.5	77.5	78.0
SNB (°)	80.1 ± 3.9	84	81	80.2
ANB (°)	2.7 ± 2.0	−6.5	−3.5	−2.2
Wits (mm)	−4.5 ± 3.0	−12.8	−15	−4.5
Go‐Me	71.0 ± 3.0	79.0	79.0	79.0
S‐Na	71.0 ± 3.0	69.0	69.0	69.0
Go‐Me/S‐Na	1.0	0.87	0.87	0.87
Vertical				
SN‐MP (°)	32.5 ± 5.6	29.5	33.2	36.0
FH‐MP (°)	26.0 ± 4.5	23.6	25.0	28.0
S‐Go/N‐Me (%)	63.5 ± 1.5	68.0	65.2	65.7
LFH (%)	55.0 ± 2.0	55.6	56.9	58.0
Saddle angle (°)	123.0 ± 5.0	122.5	131.0	119.0
Articular angle (°)	143.0 ± 6.0	142.5	140.0	151.0
Gonial angle (°)	130.0 ± 7.0	124.5	123.0	123.5
Sum (°)	396.0 ± 6.0	389.5	394.0	393.5
Dental				
U1‐SN (°)	105.7 ± 6.3	108.2	108.5	112.0
U1‐NA (mm)	5.0 ± 2.0	4.5	5.0	8.0
IMPA (°)	92.6 ± 7.0	72.0	79.5	72.5
L1‐NB (mm)	6.0 ± 2.5	0.7	0	1.0
U1/L1 (°)	125.4 ± 7.9	149.1	145.5	141.0
Soft tissue				
Nasolabial angle (°)	110 ± 2.5	69.0	73.0	79.0
E‐Line—upper lip (mm)	−1.10 ± 2.15	−5.0	−6.0	−6.0
E‐Line—lower lip (mm)	0.80 ± 2.03	−3.0	−4.5	−5.0
TVL—upper lip (mm)	4.0	6.0	5.0	5.3
TVL—lower lip (mm)	2.0	7.8	4.5	4.0
TVL—soft tissue B point (mm)	−5.0	3.5	0	−2.0
TVL—soft tissue Po popopoint (mm)	−3.0	6.0	4.0	1.8

To achieve the perfect treatment objective, the optimal plan of combination of the orthodontic treatment and orthognathic surgery was advised by the orthodontist. But the plan was refused, and the orthodontic compromised treatment plan was chosen by the patient.

**Figure 4:**
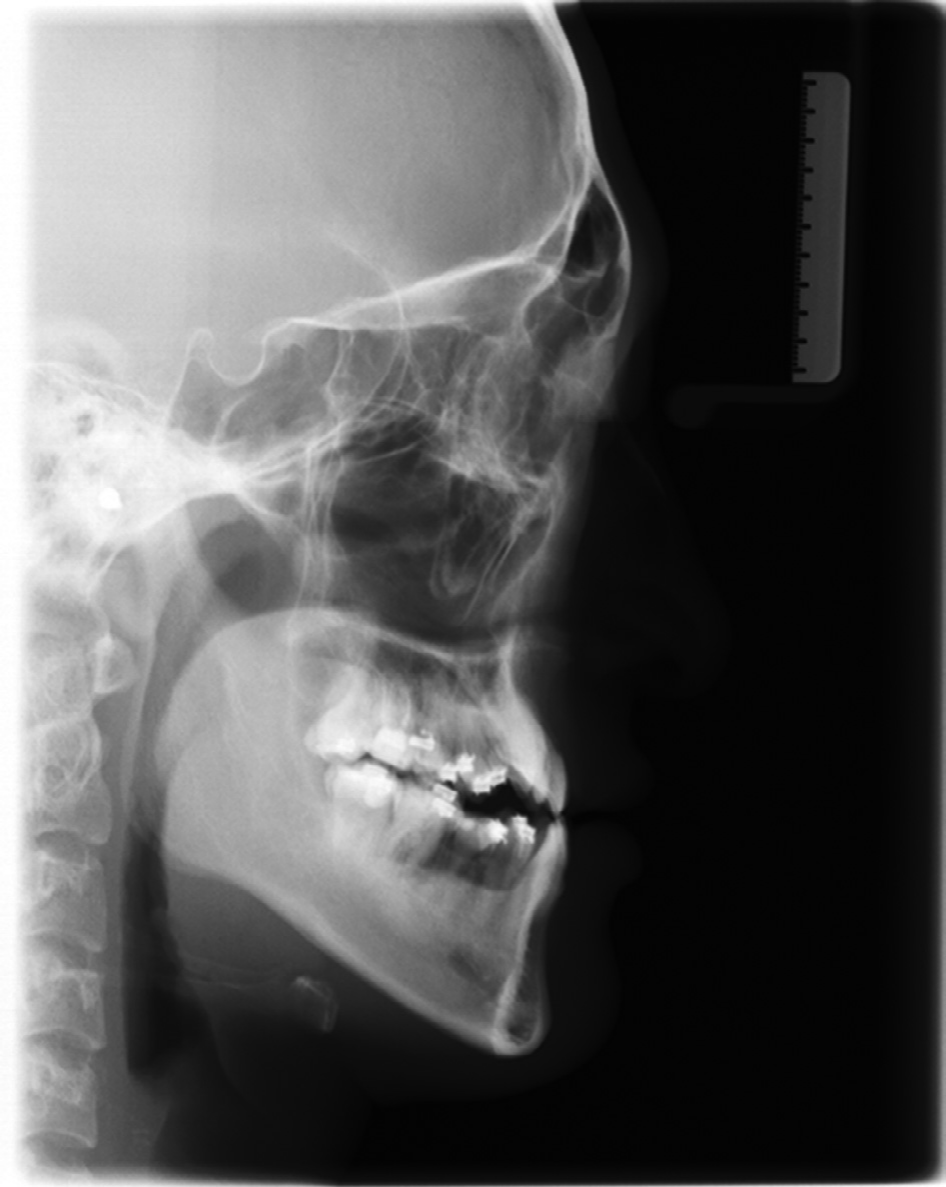
The lateral cephalogram when the functional shift of the mandible was eliminated and the incisors exhibited the edge‐to‐edge relationship.

**Figure 5:**
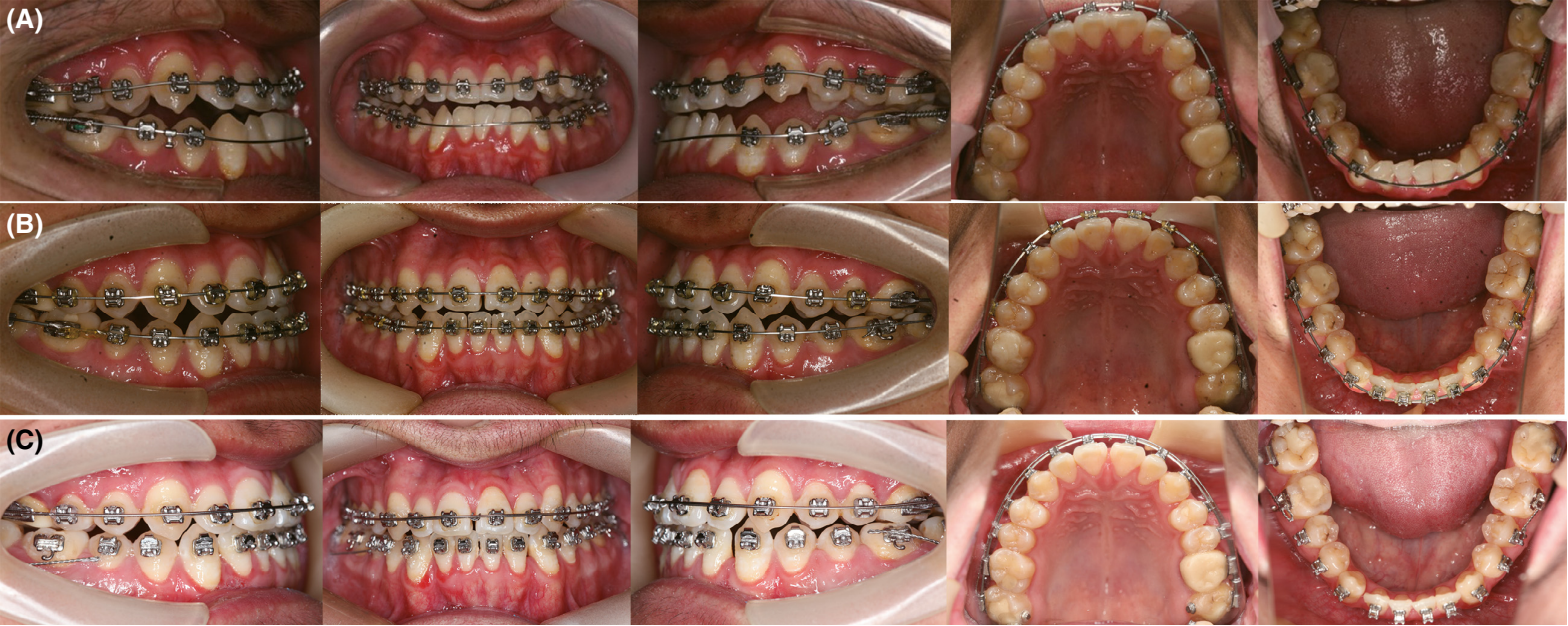
Treatment processes. (A) The functional shift of the mandible was eliminated. (B) All the brackets were bonded to align and level the dentition. (C) The micro‐implants were applied continuously to distalize the mandibular dentition.

Initially, an occlusion splint was placed in the maxillary arch and the preadjusted appliance with 0.022 × 0.028‐in slots was boned on the mandibular posterior teeth. The combination of maxillary occlusion splint and Class III elastics (1/4 inch, 3.5 oz; Ormco) eliminated the intermaxillary locked bite and the functional shift of the mandible (Fig. 4). The interproximal enamel reduction was operated on the mandibular posterior teeth to help adjust the sagittal relationship (Fig. 5A).

After 6 months, the maxillary teeth and mandibular anterior teeth were boned (Fig. 5B). The micro‐implant (2.0 × 9.0 mm; Cibei, Ningbo, China) were placed in the mandibular buccal shelf region between the first molar and second molar. The elastic materials with a continuous force of about 200 g on one side were applied from the neck of the micro‐implant to the canine to distalize the mandibular dentition (Fig. 5C).

**Figure 6:**
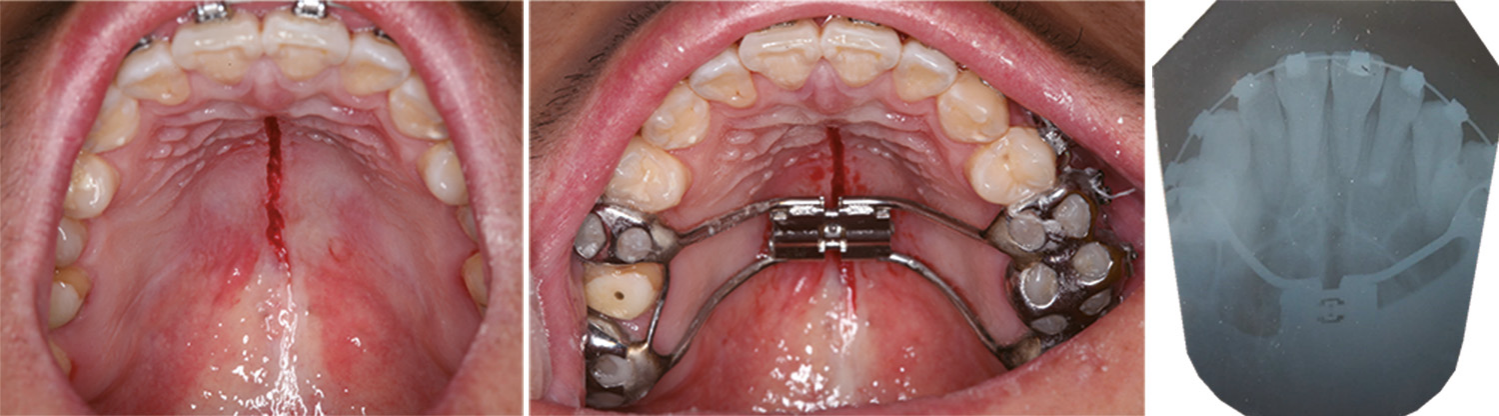
The mid‐palatal cut, tooth‐borne hyrax expander, and the occlusal radiograph after RME.

At the 24th month, the sagittal problem was almost solved. It was found that the serious lateral tipping occurred on the maxillary posterior teeth to camouflage the deficient width of the maxilla (Fig. 5A). Therefore, the surgically assisted rapid palatal expansion (SARPE) was necessary to widen the maxilla and improve the inclination of maxillary posterior teeth. The mid‐palatal suture was split which was operated with a bur by a surgeon. The cut spreads from the incisive papilla to the rear end of the hard palate with the depth of the two‐thirds thickness of the palatal cortex. The customized rapid maxillary expansion (cRME) was used, which covered on the maxillary bilateral posterior teeth except the right first molar because of the falling off of the temporary crown. The cRME was activated by turning the screw twice a day (0.5 mm per day) for 12 days, and then, the screw was turned back twice a day and was activated again in the same way. The mid‐palatal suture opening was confirmed by an occlusal radiograph. The screw was locked with the resin and kept for 3 months as a stabilizer (Fig. 6). The orthodontic treatment lasted for 34 months. The Hawley‐type removable appliances were advised to wear 24 months, followed by 12 months of nighttime wear.

**Figure 7:**
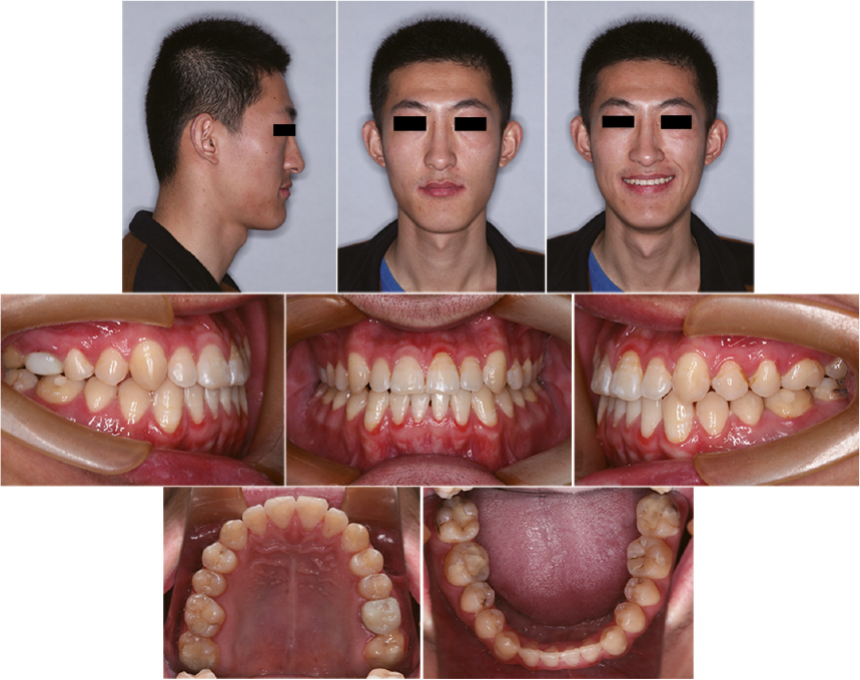
Post‐treatment extra‐oral and intra‐oral photographs.

**Figure 8:**
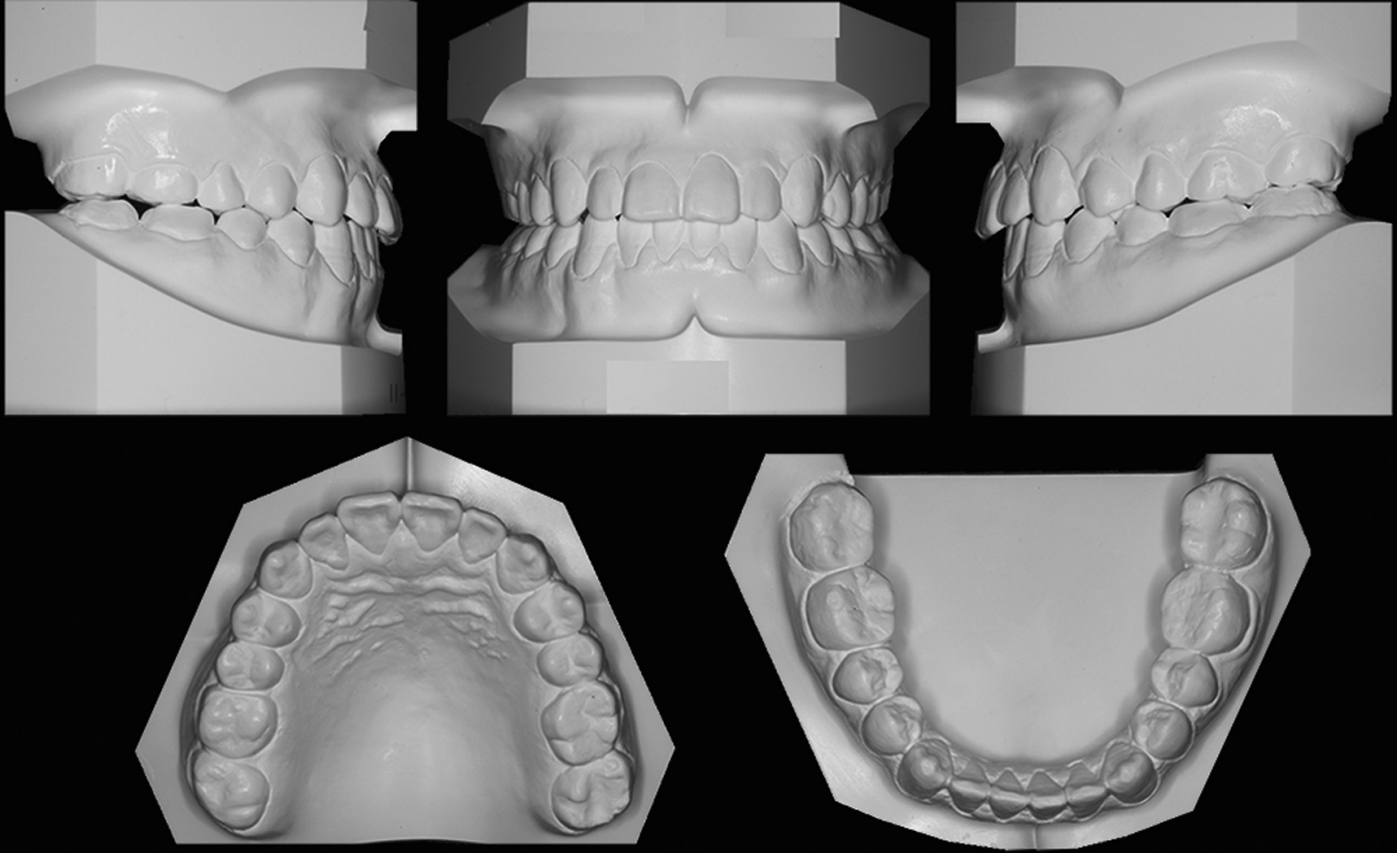
Post‐treatment dental casts.

**Figure 9:**
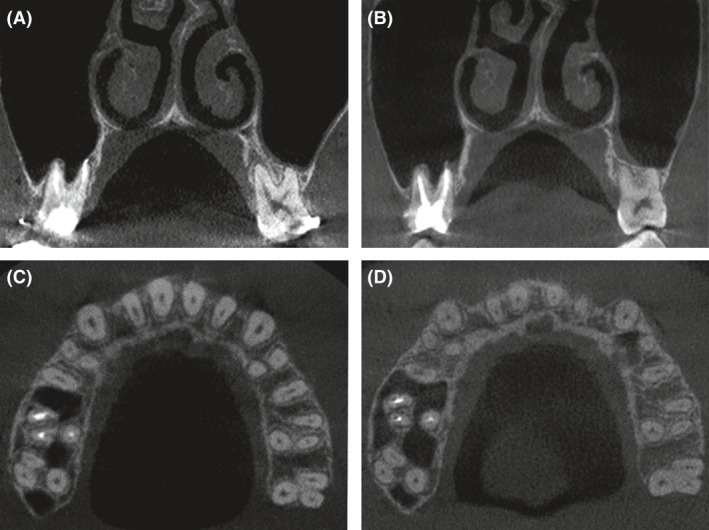
Cone‐beam computed tomography (CBCT) images. (A) CBCT image of the coronal section was taken before the RME to indicate the inclination of the maxillary first molars. (C) CBCT image of the axial section was taken before the RME at the one‐third part close to the root apex of the maxillary first molars. (B and D) The image of the coronal section was taken after the orthodontic treatment.

The post‐treatment photographs showed favorable facial changes. The deviated chin was improved, and the smile arc was enhanced dramatically with the maxillary incisal edges and cusp tips running along with the curvature of the lower lip. The lower lip was retracted, and the labiomental fold was deepened (Fig. 7). Intra‐orally, the dentition crowding was eliminated and the cross‐bite of anterior and posterior teeth was corrected. The Class I molar and canine relationship was achieved. The transverse dimension of the dental arch changed significantly after treatment. Although 3.0 mm width expansion at the maxillary first molar, the roots of first molars were in the middle of alveolar bone with good inclination (Figs 8 and 9).

**Figure 10:**
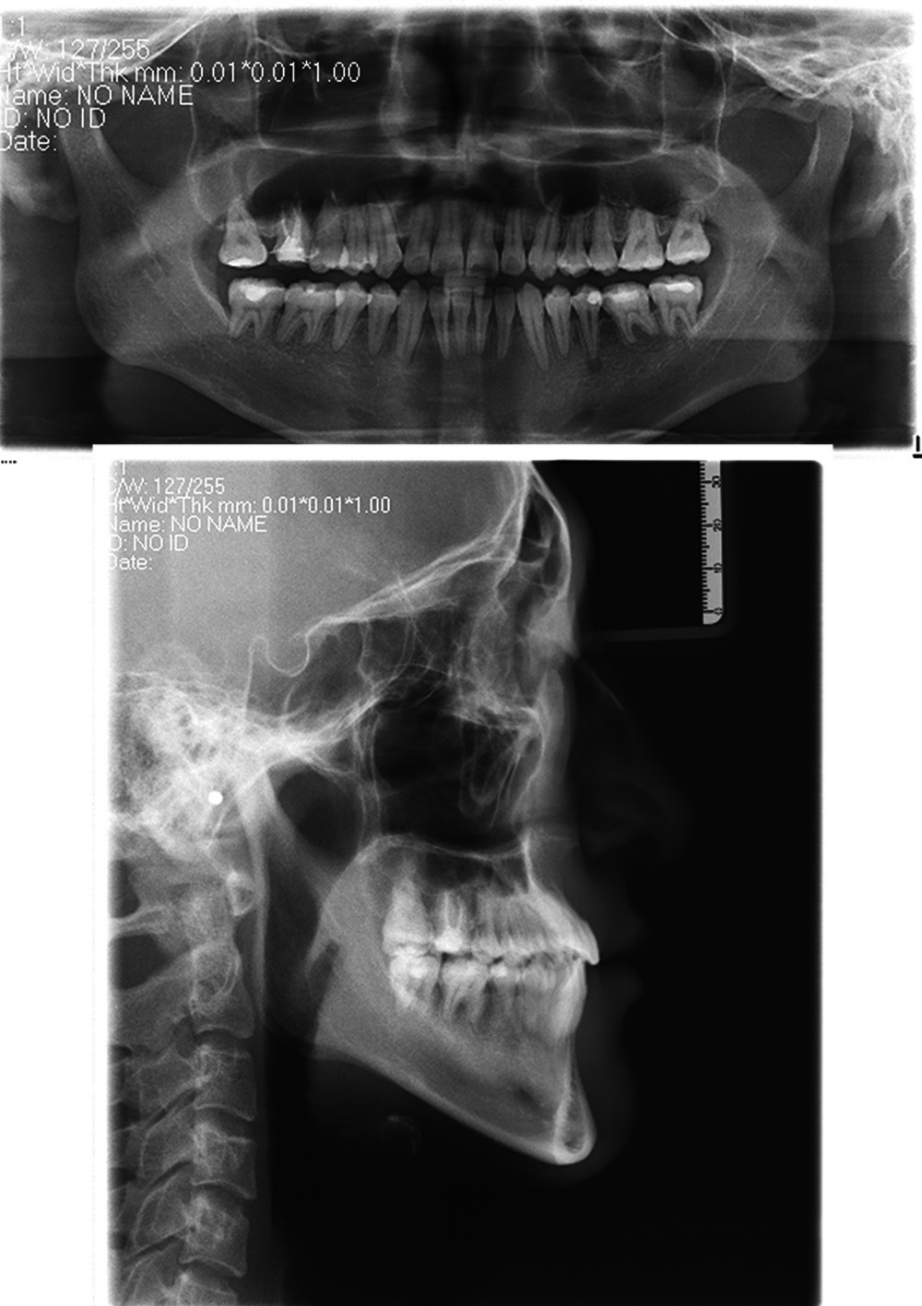
Post‐treatment panoramic radiograph and lateral cephalogram.

**Figure 11:**
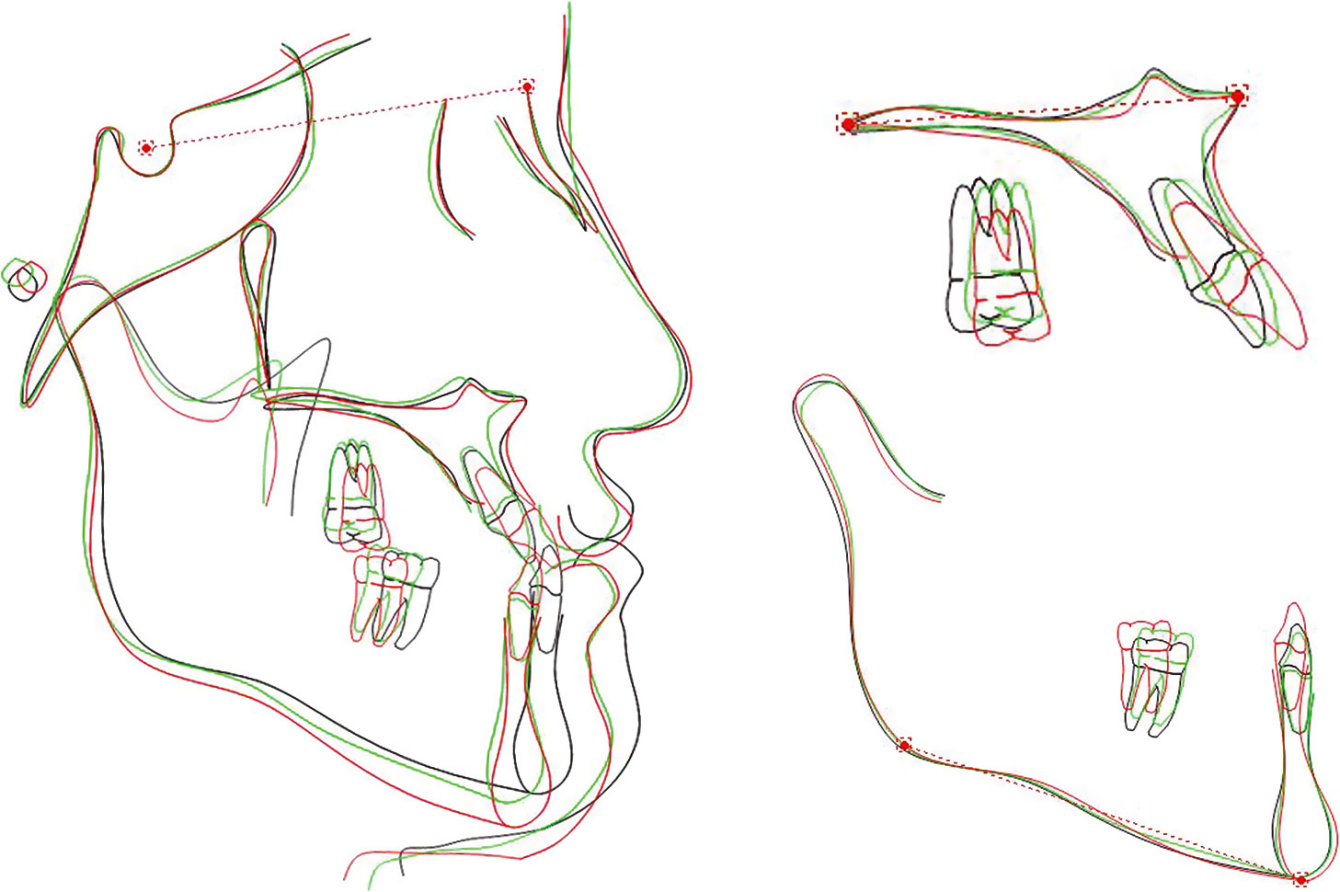
Superimpositions of the cephalometric tracings before, during and after treatment (black line, pretreatment; green line, during treatment when the functional shift of the mandible was eliminated; red line, post‐treatment).

The post‐treatment panoramic radiograph showed healthy alveolar bone and good root parallelism. From the cephalometric analysis, the SNB angle was reduced from 84° to 80.2°, which contributed to the increasing of ANB angle and wits appraisal value. In terms of vertical dimension, the value of SN‐MP and LFH was increased. The maxillary incisors were proclined, and the inclination of the mandibular incisors was almost unchanged. The elimination of the functional shift of the mandible led to a 3.8 mm retraction of lower lip, 5.5 mm retraction of the soft tissue B point, and 4.2 mm retraction of the pogonion point of the soft tissue in relation to the true vertical line (TVL) (Figs 10 and 11; Table 1).

## Discussion

In our patient, the maxillary occlusal splint was worn initially to eliminate the intermaxillary locked bite and the discrepancy of centric occlusion and centric relation in the sagittal dimension. Such the mandible was guided to a stable position, and the incisors exhibited an edge‐to‐edge relationship, which reduced the difficulty level of the solving the sagittal discrepancy problems. Therefore, it is noteworthy for clinicians that the proper orthodontic diagnosis and treatment should start from the mandibular position when the functional shift of the mandible was eliminated 12.

The literature recorded that the distalized force originating from the micro‐implants to the canine brackets tended to produce an outward moment, which led to the increasing of the arch width and the crown buccal torque of the posterior teeth 10. Thereby, the proper size and stiffness of arch wires should be considered and we added the extra crown lingual torque and constricted posteriorly on the arch wires to antagonize the unwanted side effects. Although temporary skeletal anchorage device is a viable modality to move mandibular molars distally, the distal en‐mass movement causes a distal tipping movement of the posterior teeth and the stability issue rewards attention 13, 14. The posterior teeth of this patient were with less distal tipping according to the post‐treatment cephalometric radiograph. The moderate arch length discrepancy without extraction in this patient was solved by the distalization together with minor interproximal enamel reduction in the mandibular posterior teeth to avoid the excessive molar distalization that contributed to the post‐treatment stability.

The maxillary expansion is usually a decisive advantage in the treatment of skeletal Class III malocclusion, and it could increase the arch perimeter and the stability obtaining from the proper overjet of the buccal segments 15, 16. In our patient, 3 mm space was gained from maxillary expansion and 5 mm from incisors proclination (2.5 mm labially) to correct the 8.0 mm discrepancy of the maxillary arch. For patients in adulthood, the mid‐palatal suture tends to be synostosed, and therefore, the SARPE may be advised to provide greater transverse expansion movement. It was suggested by Timms to intervene surgically only with mid‐palatal split to free the maxillae for patients between 25 and 30 years 17. Of course, the extent of the skeletal mature is more important than the chronological age. According to the extent of skeletally mature of this patient, the SARPE involving the mid‐palatal split was applied.

The tooth‐borne hyrax expander was customized metal framework that is less irritating to the palatal mucosa and surgical cut. The activation rate of the expansion was 0.5 mm per day, which was applied to avoid the premature consolidation. We used the expansion–constriction protocol to simulate the distraction osteogenesis, which stretched the soft callus in the craniofacial sutures to growing more new bone 18, 19. In our case, 3.0 mm width expansion was gained at the maxillary first molar with the enough the buccal bone cortex and the proper teeth inclination, which confirmed again that the SAPRE could be kind to the periodontal status and the teeth movement.

The clockwise rotation of the mandible can be noticed from the cephalometric superimposition, which is one of the effects brought by the RME 20. The backward rotation of the mandible was a necessary strategy in the compromised treatment of the Class III malocclusion to coordinate the maxillary width and occlusion because of the small and back maxilla 21. It leads to the backward movement of the chin, deepening the labiomental fold and improving the lower third facial profile, especially for patients with low‐angle or average‐angle facial pattern.

Lastly, the concept of the periodontal biotype was introduced 22 and the thickness of the gingival tissue should be assessed and monitored before and during orthodontic treatment. The thin alveolar housing restricted the amount of the mandibular incisor retraction. If the mandibular anterior teeth were retracted or lingually inclined too much, the risk of the dehiscence may occur. The biologic limitation of the maxillary posterior teeth movement when expansion should also be cautioned. If the periodontal integrity becomes worse during the orthodontic camouflage treatment, the combination of the orthodontic treatment and orthognathic surgery may be a better choice.

## Conclusions

This case report indicates that for an adult patient with a moderate Class III malocclusion and the functional shift of the mandible, the forward functional displacement of the mandible should be eliminated and the mandibular micro‐implants anchorage can be applied to distalize the mandibular dentition. Besides, the surgically assisted rapid maxillary rapid expansion with the expansion–constriction protocol was effective for the nongrowing patients. All the relevant factors affecting the gnathostomatic system including mandibular stability and periodontal health should be considered, and then, the function, esthetic, and stability could be optimized in the long run.

## Ethics Approval and Consent to Participate

The presented case report is in accordance with the ethical standards of the institutional and/or national research committee and with the 1964 Helsinki Declaration. Informed consent for participation was obtained from the patient presented. As no experimentation with human subjects was performed, no approval by an ethics board was required.

## Consent for Publication

Informed consent for publication of this article and its contents was obtained from the patient presented.

## Availability of Data and Materials

Raw data and complete patient documentation are available from the author Jing Guo upon request and with patient consent, but are not provided publically due to consideration of medical confidentiality.

## Conflict of Interest

The authors report no financial or other conflict of interest relevant to this article, Furthermore, no part of this article has been published before or is considered for publication elsewhere. It has been approved by all authors and the affiliated institution.

## Authorship

JG: contributed to the diagnostics, design, and coordination of the study. PPL, HC, and XXS: performed the treatment reported and provided clinical–radiological photograph and the documentation of the plaster models. PPL: drafted the manuscript and tables. JG: revised the manuscript. All authors: read and approved the final manuscript.
